# Métastase costale révélant un carcinome épidermoïde de l’œsophage

**DOI:** 10.11604/pamj.2017.26.23.9466

**Published:** 2017-01-18

**Authors:** Mamadou Ngoné Gueye, Gnagna Diouf, Daouda Dia, Awa Boye, Thierno Fall, Jean Louis Diémé, Oumar Ba, Mouhamadou Mbengue

**Affiliations:** 1 Service d’Hépato-Gastroentérolgie, Hôpital Géneral de Grand Yoff, (HOGGY) Dakar, Sénégal

**Keywords:** Cancer, œsophage, metastases, côtes, Cancer, esophagus, metastasis, ribs

## Abstract

Le cancer de l'œsophage est une tumeur de mauvais pronostic. Sa gravité est liée au retard de diagnostic qui se fait le plus souvent au stade de métastase en Afrique. Les métastases costales de ce cancer sont rares. Nous rapportons un cas de carcinome épidermoïde du bas œsophage avec métastases costales lytiques chez une patiente sénégalaise de 38 ans. Il s’agissait de madame TD, âgée de 38 ans admise pour une tuméfaction douloureuse de l’hémithorax droit dans un contexte d’amaigrissement. La patiente signalait par ailleurs une dysphagie d’allure mécanique évoluant depuis 4 mois qui n’avait pas motivé de consultation. L’examen objectivait un mauvais état général, une tuméfaction dure, sensible mesurant 3 cm de grand axe, siégeant sur la face antérolatérale de l’hémithorax droit en regard de la 5^ème^ côte. Les examens biologiques mettaient en évidence une anémie normochrome normocytaire avec un taux d’hémoglobine à 9,4 g/dl, un syndrome inflammatoire biologique non spécifique et une hypercalcémie (calcémie corrigée= 107 mg/l). L’endoscopie oeso-gastroduodénale objectivait une lésion ulcéro bourgeonnante sténosante à 32 cm des arcades dentaires. L’examen anatomopathologique des biopsies mettait en évidence un carcinome épidermoïde moyennement différencié. La tomodensitométrie thoraco-abdomino pelvienne montrait en plus de la tumeur œsophagienne, une lyse osseuse de l’arc antérieure de la 5^ème^ côte, des nodules pulmonaires carcinomateuses et un épanchement pleural bilatéral. La ponction exploratrice du liquide pleural ramenait un liquide sérohématique et l’examen cytologique de ce liquide objectivait des cellules carcinomateuses. Le diagnostic de carcinome épidermoïde du bas œsophage avec métastases costales, pleurales et pulmonaires était retenu et un traitement palliatif instauré. L’évolution était marquée par le décès de la patiente survenu 3 mois après la réalisation de la gastrostomie, dans un tableau de détresse respiratoire. L’originalité de cette observation tient au siège atypique des métastases de ce cancer de l’œsophage; mais aussi le terrain de survenue de cette tumeur. Le cancer de l’œsophage du sujet jeune est un problème majeur en Afrique. Le challenge est de déterminer ses facteurs de risque afin de prévenir sa survenue.

## Abstract (en)

Esophageal cancer is associated with poor prognosis. Its severity is linked to delayed diagnosis which is most often made once a cancer has metastasized, in Africa. Costal metastases are rare. We report a case of a 38-year old Senegalese patient with squamous cell carcinoma of the lower esophagus with lytic metastases to the ribs. Mrs. TD, aged 38, was admitted with painful swelling in right hemithorax associated with weight loss. The patient also reported mechanical dysphagia evolving during 4 months which had not motivated consultation. Clinical examination showed a poor general condition, a hard, sensitive swelling measuring 3 cm along its longer axis, located on the anterolateral surface of the right hemithorax at the level of the 5^th^rib. Biological examinations showed normocytic normochromic anemia with hemoglobin level of 9.4 g/dl, non-specific biological inflammatory syndrome, and hypercalcemia (corrected calcium = 107 mg/l. Oesogastroduodenal endoscopy showed a ulcerative, budding, stenotic lesion 32 cm from the dental arches. Anatomopathological examination of the biopsies revealed moderately differentiated squamous cell carcinoma. In addition to oesophageal tumor, thoracoabdominal-pelvic computed tomography showed bone lysis involving the anterior arch of the 5^th^ rib, carcinomatous pulmonary nodules and bilateral pleural effusion. Pleural fluid aspiration through an exploratory needle showed serohematic fluid and the cytological examination of this fluid objectified carcinomatous cells. The diagnosis of squamous cell carcinoma of the lower esophagus with rib, pleural and pulmonary metastases was retained and palliative treatment was initiated. The evolution was marked by the death of the patient 3 months after gastrostomy, within a context of respiratory distress. The originality of this observation is related to the atypical seat of metastases of this cancer of the esophagus as well as the risk factors of this tumor. Cancer of the esophagus in young adults is a major problem in Africa. The challenge is to determine its risk factors in order to prevent its occurrence.

## Introduction

Le cancer de l´œsophage est une tumeur de mauvais pronostic. Sa gravité est liée au retard de diagnostic qui se fait le plus souvent au stade de métastase en Afrique [1]. Les principaux sièges de localisations secondaires sont le foie, les poumons, la plèvre et les glandes surrénales. Les métastases costales de ce cancer sont rares [2]. Nous rapportons un cas de carcinome épidermoïde du bas œsophage avec métastases costales lytiques chez une patiente sénégalaise de 38 ans.

## Patient et observation

**Figure 1:**
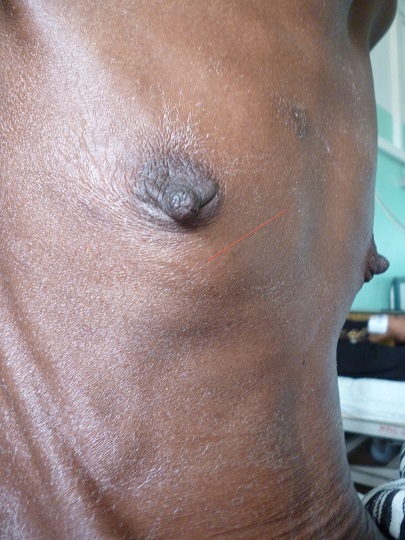
Tuméfaction de la face antérolatérale de l’hémithorax droit en regard de la 5^ème^ côte

Il s’agissait de madame TD, âgée de 38 ans admise pour une tuméfaction douloureuse de l’hémithorax droit dans un contexte d’amaigrissement (15kg en 3 mois). La patiente signalait par ailleurs une dysphagie d’allure mécanique évoluant depuis 4 mois qui n’avait pas motivé de consultation. Elle ne consommait pas de tabac ni d’alcool et ne présentait aucun antécédent pathologique particulier. L’examen objectivait un mauvais état général (Indice de performance de l’OMS à 3), une tuméfaction dure, sensible mesurant 3 cm de grand axe, siégeant sur la face antérolatérale de l’hémithorax droit en regard de la 5^ème^ cote (Figure 1).

**Figure 2:**
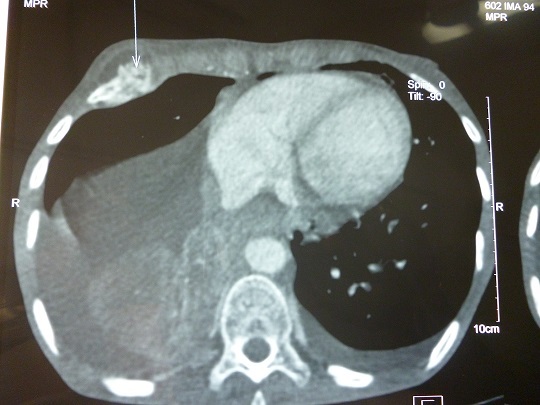
Lyse osseuse de l’arc antérieure de la 5^ème^ côte

Les examens biologiques mettaient en évidence une anémie normochrome normocytaire avec un taux d’hémoglobine à 9,4 g/dl, un syndrome inflammatoire biologique non spécifique et une hypercalcémie (calcémie corrigée= 107 mg/l). L’ionogramme sanguin, la fonction rénale de même que la fonction hépatique étaient normaux. L’endoscopie oeso-gastroduodénale objectivait une lésion ulcéro bourgeonnante sténosante à 32 cm des arcades dentaires. L’examen anatomopathologique des biopsies mettait en évidence un carcinome épidermoïde moyennement différencié. Dans le cadre du bilan d’extension, la tomodensitométrie thoraco-abdomino pelvienne montrait en plus de la tumeur œsophagienne, une lyse osseuse de l’arc antérieure de la 5^ème^ côte (Figure 2), des nodules pulmonaires carcinomateuses et un épanchement pleural bilatéral. La ponction exploratrice du liquide pleural ramenait un liquide sérohématique et l’examen cytologique de ce liquide objectivait des cellules carcinomateuses.

Le diagnostic de carcinome épidermoïde du bas œsophage avec métastases costales, pleurales et pulmonaires était retenu et un traitement palliatif instauré. Ce dernier consistait à la mise en place d’une gastrostomie d’alimentation et la prescription d’antalgiques et de biphosphonates. L’évolution était marquée par le décès de la patiente survenu 3 mois après la réalisation de la gastrostomie, dans un tableau de détresse respiratoire.

## Discussion

Les métastases costales des cancers de l’œsophage sont rares. Au Japon, dans une série de 44 patients présentant un cancer de l’œsophage, 2 cas de métastases costales ont été rapportés; tandis que dans la série de Jennings, 11 cas de métastases costales ont été répertoriés sur 719 cancers œsophagiens colligés [3, 4].

La côte est la troisième localisation osseuse de lésions secondaires après le rachis et le fémur. Ces lésions peuvent être uniques ou multiples. Les métastases costales dépassent rarement 5cm et sont souvent focales. L’aspect en imagerie est variable, il s’agit le plus souvent d’une lésion ostéolytique comme c’était le cas dans notre observation, rarement condensante. Les métastases ostéolytiques s´observent lorsque la tumeur secrète la vitamine D *like*, les prostaglandines PTHrP ou encore des cytokines pouvant être à l´origine d´une formation d´ostéoclastes, il s´agit alors d´interleukine 1 et TNF [5]. Ces métastases ostéolytiques sont responsables d’une hypercalcémie pouvant mettre et en jeu le pronostic vital à court terme.

Hormis la rareté de cette localisation secondaire, le terrain sur lequel est survenu ce cancer est à souligner. Il s’agissait d’une patiente jeune, ne présentant pas les facteurs de risque classique du carcinome épidermoide de l’œsophage à savoir le tabagisme, l’alcoolisme, des antécédents de syndrome de plummer vinson ou d’oesophagite caustique. En Asie et en occident, la survenue du cancer de l’œsophage avant 40 ans reste exceptionnelle [6]. En Afrique, plusieurs auteurs ont rapporté la survenue de cancer de l’œsophage chez des sujets jeunes. Dans la série de Dia et al, 70% des patients étaient âgés de moins de 60 ans et 13,2% de moins de 30 ans [7]. Ce profil d’âge était proche de celui noté par Parker *et al*. Dans l’Ouest du Kenya avec 6,3 % de patients âgés de moins de 30 ans [8]. Des zones d’ombres persistent concernant les facteurs étiologiques de ces cancers de l’œsophage chez les sujets jeunes en Afrique.

Le pronostic de ce cancer reste sombre. Du fait de la non disponibilité des examens endoscopiques dans certaines régions, le diagnostic est souvent tardif, au stade de métastase comme c’était le cas chez notre patiente. Les localisations secondaires osseuses accentuent la mortalité par l’hypercalcémie majeure qu’elles peuvent entrainer. La prise en charge de cette complication métabolique est particulièrement difficile en Afrique sub saharienne à cause de la cherté des diphosphonates.

## Conclusion

L’originalité de cette observation tient au siège atypique des métastases de ce cancer de l’œsophage; mais aussi le terrain de survenue de cette tumeur. Le cancer de l’œsophage du sujet jeune est un problème majeur en Afrique. Le challenge est de déterminer ses facteurs de risque afin de prévenir sa survenue. La pratique de l’endoscopie digestive doit être vulgarisée afin que cet examen soit accessible à toutes les populations de l’Afrique sub saharienne, permettant ainsi de réduire les délais diagnostiques et de mieux prendre en charge ces cancers.
